# QTL mapping for early heading of ‘Haruka Nijo’, the leading barley (*Hordeum vulgare* L.) cultivar in the Kyushu region of Japan

**DOI:** 10.1270/jsbbs.25054

**Published:** 2026-04-02

**Authors:** Tomohiko Sugita, Takuji Tonooka, Shuichi Fukuoka, Yuki Nakano, Yusuke Ban, Tsuyoshi Tanaka, Junichi Tanaka

**Affiliations:** 1 National Agriculture and Food Research Organization (NARO) Western Region Agricultural Research Center (Kinki, Chugoku and Shikoku Regions), 6-12-1 Nishifukatsu-cho, Fukuyama, Hiroshima 721-8514, Japan; 2 Graduate School of Science and Technology, University of Tsukuba, 2-1-2 Kannondai, Tsukuba, Ibaraki 305-8518, Japan; 3 NARO Kyushu Okinawa Agricultural Research Center, 496 Izumi, Chikugo, Fukuoka 833-0041, Japan; 4 Ministry of Agriculture, Forestry and Fisheries, 1-2-1 Kasumigaseki, Chiyoda-ku, Tokyo 100-8950, Japan; 5 NARO Institute of Crop Science, 2-1-2 Kannondai, Tsukuba, Ibaraki 305-8518, Japan; 6 NARO Central Region Agricultural Research Center (Joetsu Research Station), 1-2-1 Inada Joetsu, Niigata 943-0193, Japan; 7 NARO Research Center for Advanced Analysis, 2-1-2 Kannondai, Tsukuba, Ibaraki 305-8518, Japan; 8 NARO Headquarters, 3-1-1 Kannondai, Tsukuba, Ibaraki 305-8517, Japan

**Keywords:** barley, heading date, early heading, ‘Haruka Nijo’, quantitative trait loci (QTLs)

## Abstract

Optimizing heading date to suit local conditions is key to maximizing yield potential. ‘Haruka Nijo’, a two-rowed barley (*Hordeum vulgare* L.) cultivar developed for the Kyushu region of Japan, is early-heading and has superior yield performance compared to the standard cultivar ‘Nishinohoshi’. To identify genomic regions associated with early-heading in ‘Haruka Nijo’, we conducted analysis of quantitative trait loci (QTLs) using recombinant progeny of ‘Haruka Nijo’ × ‘Nishinohoshi’ (heading date difference: 2.4–6.0 days). A stable QTL, designated *QHD.HN-5H*, was detected near the centromere of chromosome 5H. This QTL explained 30.3–46.1% of the phenotypic variance and consistently conferred 2–5 days earlier heading across three seasons. Pedigree analysis indicated that the *QHD.HN-5H* region in ‘Haruka Nijo’ likely originated from the Tohoku six-rowed cultivar ‘Haganemugi’ and was probably co-introduced into Kyushu cultivars together with the Barley yellow mosaic virus resistance gene *rym3*. Whole-genome sequencing and Gene Ontology analysis identified non-synonymous differences between ‘Haruka Nijo’ and ‘Nishinohoshi’ in five heading-related genes within the QTL region. Four of these genes shared identical genotypes between ‘Haganemugi’ and ‘Haruka Nijo’, supporting their candidacy. These findings provide new breeding tools to adapt the heading date of barley to the climate and cultivation environment.

## Introduction

Barley (*Hordeum vulgare* L.) is the fourth most produced cereal crop and is widely cultivated worldwide, from subarctic regions such as Scandinavia to tropical and subtropical regions such as North Africa, East Africa, and South Asia (FAOSTAT 2025). Optimizing its heading date is one of the most important breeding objectives for maximizing and stabilizing yields under various regional climatic conditions. In Japan, barley is commonly grown in a rice–barley rotation system in paddy fields, with rice (*Oryza sativa* L.) typically grown from June to October and barley from November to May. Barley harvesting must be completed before field preparation for rice cultivation begins. In addition, the rainy season usually starts in June, increasing the risk of pre-harvest sprouting and grain quality loss if the barley has not yet been harvested (Chono *et al.* 2015). For these reasons, early heading is a key trait for barley cultivation. This is especially important in Kyushu (in southwestern Japan), where the rainy season starts earlier than in other regions. Consequently, early heading is an important and major breeding goal for barley cultivated in the Kyushu region. However, the genetic factors underlying differences in heading date among barley cultivars in Kyushu are still unclear, and genetic tools useful for breeding early heading remain limited.

Researchers seeking a deeper understanding of heading date have identified several genes associated with this trait. In rice, heading date is controlled by complex genetic networks (Colleoni *et al.* 2024, Hori *et al.* 2016, Vicentini *et al.* 2023). Heading date in barley is primarily controlled by two factors: vernalization requirements and photoperiodic response (Fernández-Calleja *et al.* 2021). Vernalization requirements are primarily controlled by *Vrn-H1*, *Vrn-H2*, and *Vrn-H3* (Yan *et al.* 2003, 2004, 2006): the loss of any one of these three genes reduces or eliminates vernalization requirements. The three genes primarily responsible for photoperiodic response in barley are *Photoperiod-H1* (*Ppd-H1*) (Turner *et al.* 2005), *PHOTOPERIOD-H2* (*Ppd-H2*) (Kikuchi *et al.* 2009), and *PHYTOCHROME C* (*HvPhyC*) (Nishida *et al.* 2013). However, the differences in these major heading-date-related genes among barley cultivars in Kyushu have not studied in detail.

We focused on the cultivar ‘Haruka Nijo’ to identify additional genetic factors related to heading date. ‘Haruka Nijo’ is a non-malting two-rowed barley cultivar released in 2012 for the Kyushu region (Kawada *et al.* 2015). ‘Haruka Nijo’ has both earlier heading and higher yield than the standard cultivar ‘Nishinohoshi’ and has excellent agricultural traits such as pre-harvest sprouting tolerance and lodging resistance. ‘Haruka Nijo’ also carries *resistance to yellow mosaic 3* (*rym3*), making it resistant to Barley yellow mosaic virus (BaYMV) strain III, whereas ‘Nishinohoshi’ is susceptible and lacks *rym3*. As a result, ‘Haruka Nijo’ cultivation has expanded in recent years, primarily in northern Kyushu, and this cultivar is widely used as a parent for breeding cultivars suitable for warmer climate region in Japan. Elucidating the genetic control of early heading in ‘Haruka Nijo’ will bring significant benefits to barley breeding in Japan.

In this study, we focused on the early heading of ‘Haruka Nijo’. We constructed recombinant inbred lines (RILs) from ‘Haruka Nijo’ and ‘Nishinohoshi’ and conducted field trials and QTL analysis concentrating mainly on heading date, which was measured during three growing seasons. As a result, we identified one stable QTL detected consistently over the three seasons on chromosome 5H. Additionally, we discovered non-synonymous variants in several candidate genes within the QTL region related to heading date through whole-genome sequencing (WGS).

## Materials and Methods

### Plant materials

#### Phenotyping and cultivation conditions

To investigate heading date variation and polymorphisms in heading-related genes among major barley cultivars in the Kyushu region, we grew four cultivars, namely, ‘Haruka Nijo’ (Kawada *et al.* 2015), ‘Nishinohoshi’ (Sasaki *et al.* 1999), ‘Harushizuku’ (Furusho *et al.* 2006), and ‘Sachiho Golden’ (Kato *et al.* 2006), in two growing seasons (2016–2017 and 2017–2018) at Kyushu Okinawa Agricultural Research Center (KARC) in Chikugo (33°12ʹ25.20ʺN, 130°29ʹ20.40ʺE). Seeds used in this study were maintained by breeding programs at KARC. These trials were conducted independently from the RIL experiments described below. Cultivation conditions are shown in Supplemental Table 1.

To perform QTL analysis for the early-heading of ‘Haruka Nijo’, we constructed an RIL population (188 lines) from a cross between ‘Haruka Nijo’ and ‘Nishinohoshi’. The RIL generations were F_6_ in 2016–2017, F_7_ in 2017–2018, and F_8_ in 2020–2021. We cultivated the RILs and their parents over three seasons in Japan: during the first two seasons (2016–2017, 94 lines; and 2017–2018, 188 lines) at KARC, and during the third season (2020–2021, 188 lines) at the Western Region Agricultural Research Center (WARC) in Zentsuji (34°13ʹ51.60ʺN, 133°46ʹ37.20ʺE). The cultivation conditions are shown in Supplemental Table 2. The temperature data were obtained from weather stations at KARC and WARC. Heading date was defined as the date when the ear began to emerge from approximately half of the plants in a plot. The heading date was indicated with March 1 considered to be day 1. Cultivation was carried out in accordance with the standard procedures routinely used at KARC and WARC. To evaluate the consistency of heading dates across replicates and seasons, scatter plots were generated and Pearson’s correlation coefficients were calculated. Simple linear regression lines were fitted to each comparison.

### Genotyping of heading-related genes in Kyushu cultivars

Six heading-related genes (*Vrn-H1*, *Vrn-H2*, *Vrn-H3*, *Ppd-H1*, *Ppd-H2*, and *PhyC*) were genotyped in four cultivars developed for the Kyushu region (‘Haruka Nijo’, ‘Nishinohoshi’, ‘Harushizuku’, and ‘Sachiho Golden’). DNA was extracted using the potassium acetate method reported in Dellaporta *et al.* (1983), and genotyping was performed as described by Nishida *et al.* (2013).

### Marker development

To construct the genetic linkage map, we developed and used one derived cleaved amplified polymorphic sequence (dCAPS) marker and 241 single-nucleotide polymorphism (SNP) assays (Standard BioTools, Tokyo, Japan), for a total of 242 markers. The selection of these markers was based on SNPs extracted according to Tanaka *et al.* (2019), to ensure even physical spacing across the genome. The primers for *HvCO3* genotyping (dCAPS marker) were HvCO3_dCAPs_BamHI_F (5ʹ-TCGATCGATCGACCATGGAAGGAGGAACGCTCATGGTCTCTCCACgGAtC-3ʹ) and HvCO3_dCAPs_R (5ʹ-AGGAAGATGAGGCGGTTCGAGAAGACCATC-3ʹ). Lowercase letters indicate mismatched bases introduced to create a restriction site. The primers for SNP genotyping system are shown in Supplemental Table 3.

### Genotyping for linkage mapping

DNA was extracted from the parents and 188 RILs using the potassium acetate method (Dellaporta *et al.* 1983). RILs (F_5_) were genotyped using the dCAPS marker for *HvCO3*. A total of 241 SNP markers were genotyped using the 96.96 Dynamic Array Integrated Fluidic Circuit (IFC) chips (Standard BioTools), following the SNPtype 96 × 96 v1 protocol (https://fluidigm.my.salesforce.com/sfc/p/#700000009DAw/a/4u0000019kFV/J5w6q0CKYdlTv5ebT5PYx8Ai2T5oGz.ClYtyMxHAWFg). For the dCAPS marker, PCR was performed in a total volume of 10 μL containing 100 ng of genomic DNA, 200 μM of each dNTP, 0.2 μM of each primer, 0.25 U of ExTaq HS DNA polymerase, and 1× ExTaq HS buffer (TaKaRa, Shiga, Japan). The PCR cycling conditions were as follows: initial denaturation at 98°C for 1 min; followed by 35 cycles of 98°C for 10 s, 65°C for 30 s, and 72°C for 30 s; followed by a final extension at 72°C for 10 min. The PCR products were digested in a total volume of 15 μL with 4.5 U of *Bam*HI (TaKaRa) and 1× K buffer (TaKaRa) at 30°C overnight. *Bam*HI digestion yielded fragments of 50 bp and 186 bp for the SNP_C allele (‘Nishinohoshi’ type) and a single 219-bp fragment for the SNP_T allele (‘Haruka Nijo’ type).

### Map construction and QTL analysis

We constructed a linkage map using RILs with MapDisto ver. 2.1.8.7 (Lorieux 2012). All settings used the defaults provided within the software package. QTL analysis was performed by composite interval mapping (CIM; Zeng 1994) using the cim() function with the R/qtl ver1.66 package (Broman *et al.* 2003). In 2016–2017 and 2017–2018, phenotypic data averaged over the two replicates were analyzed separately for each season. CIM was implemented using the Kosambi mapping function (Kosambi 1943) with a window size of 0.1 cM and three markers selected as covariates. The calculations of the logarithm of odds (LOD) were based on 1000 permutation tests at the α = 0.05 experiment-wide error rate (Doerge and Churchill 1996).

### Pedigree analysis

To identify the origin of the detected QTL for heading date (see Results), 12 varieties from the pedigree of ‘Haruka Nijo’ (Kawada *et al.* 2015) were genotyped in that QTL region. To perform the analysis, a dCAPS marker was designed for FB0333, the nearest marker to the QTL. PCR was performed in a total volume of 10 μL containing 100 ng of genomic DNA, 200 μM of each dNTP, 0.2 μM of each primer, 0.25 U of ExTaq HS DNA polymerase, and 1× ExTaq HS buffer (TaKaRa). The primers used were FB0333_dCAPS_NdeI_F1 (5ʹ-AAATTAATCTACCTCCGAAAGCAGCGATATGTCTACTTTGCTcaTA-3ʹ) and FB0333_dCAPS_R1 (5ʹ-CTCCATTGAATACTAGTGGAAGGGGAAGAACACAC-3ʹ). Lowercase letters indicate mismatched bases introduced to create a restriction site.

The PCR was performed with an initial denaturation at 98°C for 3 min; followed by 30 cycles of 98°C for 10 s, 65°C for 30 s, and 72°C for 30 s; followed by a final extension at 72°C for 3 min. The PCR products (10 μL) were digested in a total volume of 15 μL with 4.5 U of *Nde*I (New England Biolabs, Ipswich, MA, USA) and 1× CutSmart buffer (New England Biolabs) at 37°C overnight. *Nde*I digestion yielded two fragments of 152 bp and 45 bp for the SNP_T allele (‘Nishinohoshi’ type) and a single 197-bp fragment for the SNP_C allele (‘Haruka Nijo’ type).

### Whole-genome sequence analysis

Genomic DNA was extracted from barley leaves using an ISOSPIN Plant DNA kit (NIPPON GENE, Tokyo, Japan), according to the manufacturer’s instructions. Sequencing libraries were prepared using the TruSeq DNA PCR-free Library prep kit (Illumina, San Diego, CA, USA) and sequenced using the Illumina NovaSeq 6000 with paired-end 150-bp (PE150) platform by Rhelixa, Inc. (Tokyo, Japan) and Macrogen Japan Corp. (Kyoto, Japan). The raw sequence reads obtained in this study are available in the DDBJ DRA database under accession numbers DRR703870–DRR703872. Illumina raw data were preprocessed by trimmomatic-0.39 to discard low-quality and adapter sequences (-phred33 ILLUMINACLIP: TruSeq3-PE.fa:2:30:10 LEADING:15 TRAILING:15 SLIDINGWINDOW:4:15 MINLEN:32) (Bolger *et al.* 2014). Processed reads were mapped on the ‘Morex’ genome sequence (version 3) using NVIDIA Clara Parabricks v4.1.1 with the options (pbrun fq2bam --fix-mate --bwa-options="-M -T 30" --gvcf) (O’Connell *et al.* 2023). Mapping reads were processed by SAMtools (Version: 1.20) (Danecek *et al.* 2021), and three cultivars were genotyped using gatk (v4.5.0.0) (McKenna *et al.* 2010). SNP effects were assigned by SnpEff (version 4.3t) (Cingolani *et al.* 2012), only those variants categorized as “high” or “moderate” impact were retained for further analysis. Genes corresponding to the retained variants were annotated using Ensembl Plants (https://plants.ensembl.org/). Gene Ontology (GO) terms were retrieved for each gene, and genes annotated with flowering-related GO terms (e.g., “flower development”, “regulation of flowering time”) were selected for further analysis. The list of GO terms used for this selection is provided in Supplemental Table 4.

### Statistical analysis

All statistical analyses were performed in R (version 4.4.1).

For 2016–2017 (*n* = 2 plots), only descriptive statistics (mean ± SD) were summarized, and no inferential statistics were performed. For 2017–2018 (*n* = 3 plots), differences in heading date among cultivars were evaluated using ANOVA followed by Tukey’s HSD for multiple comparisons.

Correlation analysis of heading date across replicates and growing seasons was conducted using the cor() function to calculate Pearson’s correlation coefficients and *p*-values. Linear regression analysis was performed using the lm() function to evaluate the reproducibility of heading date across environments.

The relationship between heading date and genotype at FB0333, the marker nearest to *QHD.HN-5H* (see Results), was evaluated among RIL groups. ANOVA was performed using the aov() function, and multiple comparisons were conducted using the TukeyHSD() function to test for significant differences in heading date between genotypic groups.

## Results

### Heading date variation and heading-related gene polymorphisms among Kyushu barley cultivars

As the first step toward identifying genetic factors underlying heading date variation in cultivars in Kyushu, we investigated differences in heading date and major heading-related genes among the four main cultivars: ‘Nishinohoshi’, ‘Sachiho Golden’, ‘Harushizuku’, and ‘Haruka Nijo’. In 2016–2017, the order of mean heading dates (earliest to latest) was ‘Sachiho Golden’ < ‘Haruka Nijo’ < ‘Harushizuku’ < ‘Nishinohoshi’. Because the cultivars were planted in only two replicates, however, the significance of differences between heading dates could not be assessed. In 2017–2018, when three replicates were planted, ‘Haruka Nijo’ and ‘Sachiho Golden’ headed significantly earlier than ‘Nishinohoshi’ and ‘Harushizuku’. Despite differences in heading dates, genotyping results showed no differences in the major heading-related genes among these cultivars (Table 1). The heading date of ‘Haruka Nijo’, the early-heading parent of the RILs, was 2.4–6.0 days earlier than that of ‘Nishinohoshi’, the late-heading parent, across the two growing seasons.

### Distribution of heading dates in RILs

**Fig. 1.**
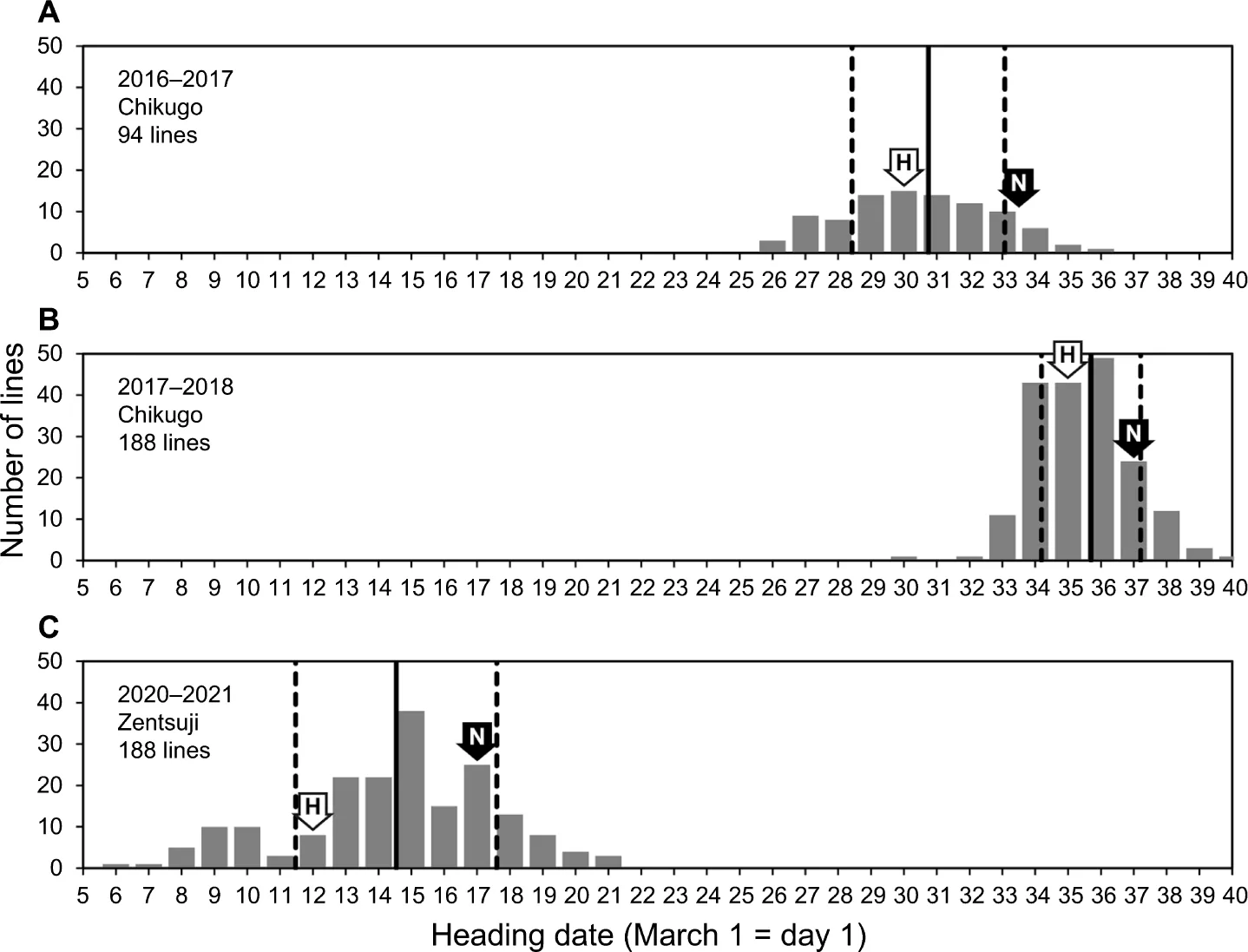
Frequency distribution of heading date in the RIL population grown in (A) 2016–2017 at Chikugo with 94 lines, (B) 2017–2018 at Chikugo with 188 lines, and (C) 2020–2021 at Zentsuji with 188 lines. The white arrow (H) indicates ‘Haruka Nijo’, and the black arrow (N) indicates ‘Nishinohoshi’. The solid lines indicates the mean of RILs, and the dotted lines indicate the mean ± SD.

The distribution of heading dates in RILs over the three seasons is shown in Fig. 1. The average temperatures in each season of the test are shown in Supplemental Fig. 1. Although the heading dates varied greatly among the three seasons, ‘Haruka Nijo’ was always 2–5 days earlier than ‘Nishinohoshi’. In 2016–2017 (Fig. 1A), heading dates (March 1 = day 1) ranged from 26 to 36 days (mean 30.7 ± SD 2.3). In 2017–2018 (Fig. 1B), the range was 30 to 40 days (mean 35.6 ± SD 1.5), the latest among the three seasons. In contrast, 2020–2021 (Fig. 1C) showed the earliest heading, ranging from 6 to 21 days (mean 14.5 ± SD 3.1). In all seasons, the distribution of heading dates in RILs showed continuous segregation, a pattern characteristic of quantitative traits. The variation among seasons was primarily attributed to differences in experimental locations and climatic conditions. The low temperature during the first part of the growing season in 2017–2018 and the high temperatures in February and March in 2020–2021 might explain the late and early heading, respectively (Supplemental Fig. 1). Despite seasonal variation, heading date measurements showed high reproducibility across years and replicates (*r* = 0.708 to 0.877, Supplemental Fig. 2).

### Genotyping and linkage map construction

A total of 241 SNP assays and 1 dCAPS marker were developed. Among them, 234 SNP markers and the dCAPS marker showed polymorphism between the RIL parents and were used to construct a linkage map, resulting in 10 linkage groups (LGs) (Supplemental Fig. 3). The total map length was 854.46 cM, with an average density of 3.67 cM per marker. While some discrepancies between genetic and physical marker orders were observed, most markers showed consistent positioning. Although the markers were selected to be evenly distributed on the physical map, the constructed linkage map consisted of multiple LGs for chromosomes 2H, 5H, and 7H. These LGs were separated by regions longer than the threshold genetic distance used for grouping markers.

### QTL analysis

**Fig. 2.**
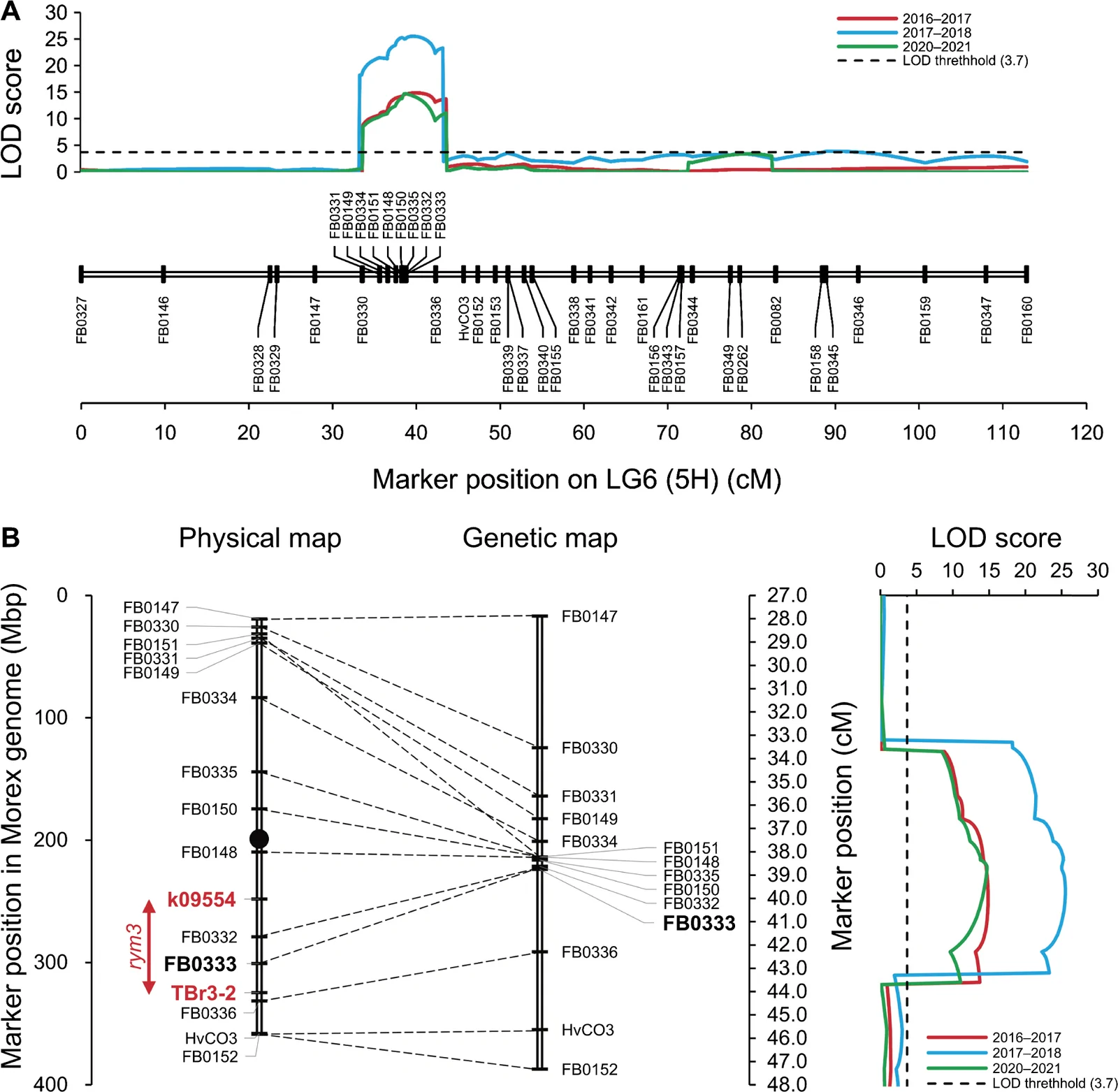
QTL mapping and marker correspondence for heading date on chromosome 5H. (A) Logarithm of odds (LOD) scores for heading date on LG6 (5H), a part of barley chromosome 5H, according to composite interval mapping. The black dotted line shows the highest LOD threshold (3.7) among the three seasons (Table 2). Genetic distances are shown in centimorgans (cM). (B) Physical map (left), genetic linkage map (middle), and LOD score profile (right) of the QTL region on chromosome 5H. Markers common to both the physical and genetic maps are connected by dotted lines. A black circle represents the approximate position of the centromere on the physical map of chromosome 5H (Navrátilová *et al.* 2022). Bold marker names indicate the markers closest to the QTL peak. Red marker names represent those linked to the *rym3* resistance gene (Haruyama *et al.* 2012). The red double-headed arrow indicates the genomic region in which *rym3* is presumed to be located, and any co-introgression with *rym3* is treated as a working hypothesis based on positional proximity rather than direct genotyping in pedigree lines.

**Fig. 3.**
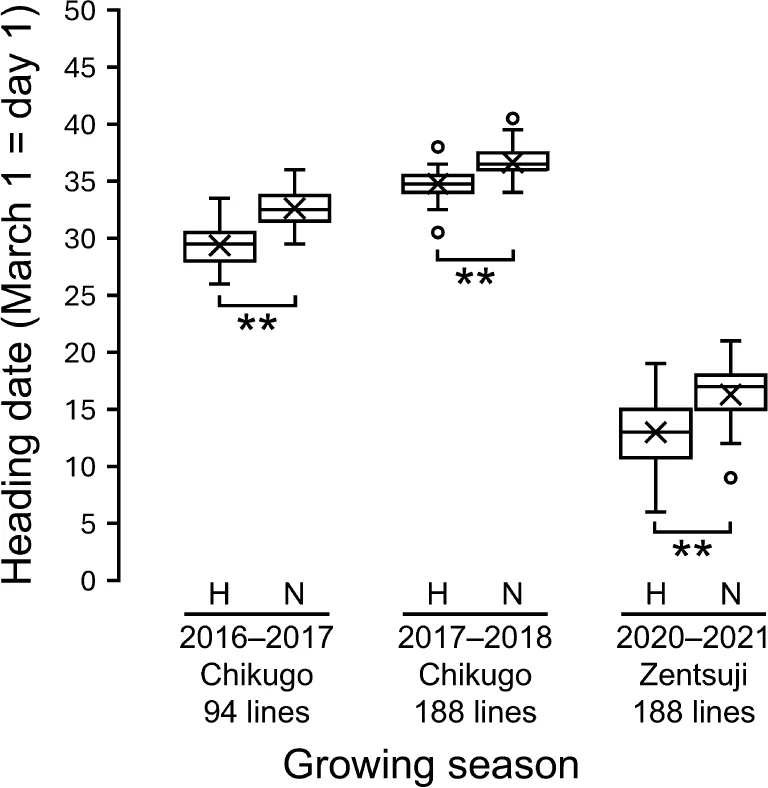
The relationship between RIL heading date and genotype of FB0333, the marker nearest to *QHD.HN-5H*. Box plots show the variation in heading dates for each season. Heading dates are expressed as cumulative days starting from March 1 (i.e., March 1 = day 1). H indicates RILs containing the FB0333 marker allele from ‘Haruka Nijo’ (early-heading parent); N indicates those containing the allele from ‘Nishinohoshi’. Each box represents the interquartile range (IQR), with the horizontal line inside indicating the median. Whiskers extend to the minimum and maximum values excluding outliers, which are shown as individual points (circles). Cross marks (×) indicate the mean values. Asterisks (**) indicate statistically significant differences within a season (Tukey’s HSD test, *p* < 0.01).

QTL analysis for heading date identified one QTL in 2016–2017, four in 2017–2018, and two in 2020–2021 (Table 2). Among these, one QTL detected at position 38.8–40.0 cM on LG6 was detected in all three seasons (Table 2, Fig. 2). This position was near the centromere of chromosome 5H (Navrátilová *et al.* 2022; Fig.2), and the QTL was named *QHD.HN-5H* (a QTL for heading date mapped in the ‘Haruka Nijo’ × ‘Nishinohoshi’ population on chromosome 5H). In all three seasons, FB0333 was the marker closest to the peak position of *QHD.HN-5H*. The peak was located between FB0147 and FB0152, corresponding to 19,352,357–358,711,871 bp in the ‘Morex’ chromosome 5H genome sequence (Fig. 2). The early-heading allele of *QHD.HN-5H* was derived from ‘Haruka Nijo’ and explained 30.3% to 46.1% of the phenotype, with additive effects of the ‘Haruka Nijo” allele ranging from –0.97 to –1.70 (Table 2). To confirm the effect of *QHD.HN-5H* in the RIL population, RILs were classified on the basis of the FB0333 genotype. RILs carrying the ‘Haruka Nijo’ genotype had significantly earlier heading dates than those with the ‘Nishinohoshi’ genotype in all three seasons, with differences ranging from 1.9 to 3.3 days (Fig. 3).

### Pedigree analysis

**Fig. 4.**
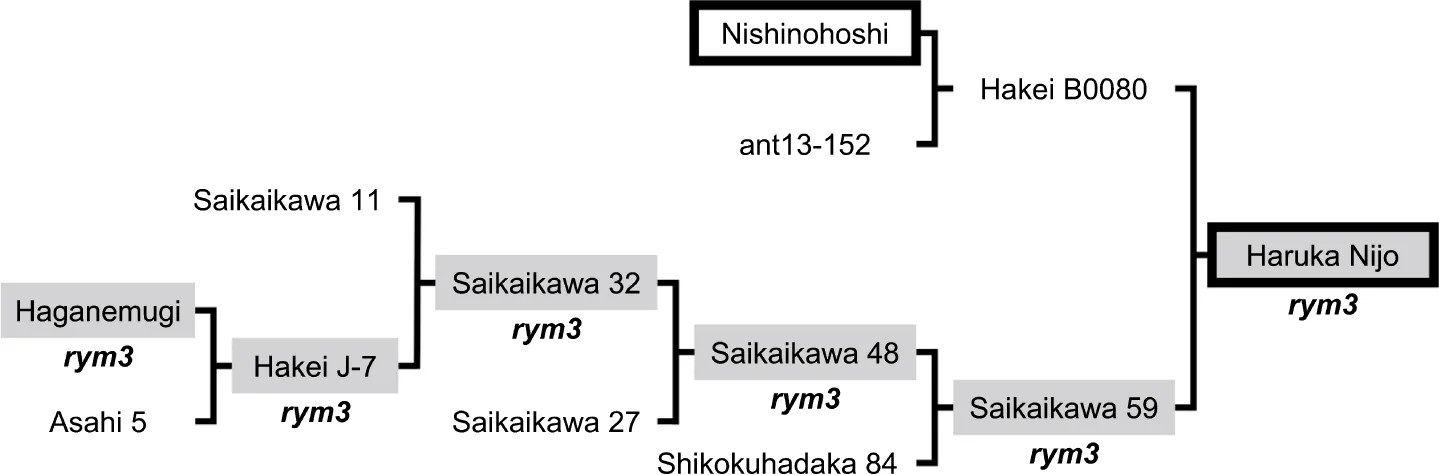
Estimated genotype of *QHD.HN-5H* in ancestors of ‘Haruka Nijo’. Bold boxes show the parents of the RIL population. Grey boxes show the cultivars containing the ‘Haruka Nijo’ allele of marker FB0333, which was closest to the QTL peak. Bold letters (*rym3*) under variety names indicate the presence of the *rym3* gene (*resistance to yellow mosaic virus 3*), which was inferred based on phenotype and positional proximity to linked markers, not on direct genotyping of *rym3*-linked markers in these pedigree lines (see Discussion).

To reveal the origin of the *QHD.HN-5H* allele from ‘Haruka Nijo’, the genotypes of varieties in the pedigree of ‘Haruka Nijo’ were determined using FB0333. The ‘Haruka Nijo’ allele of FB0333 was found in the Kyushu breeding lines ‘Saikaikawa 59’, ‘Saikaikawa 48’, ‘Saikaikawa 32’, and ‘Hakei J-7’ and their ancestor, the Tohoku six-rowed barley cultivar ‘Haganemugi’. Additionally, this genotype was associated with the BaYMV resistance gene *rym3* (Fig. 4).

### Detection of candidate genes for *QHD.HN-5H* by WGS

To search for candidate genes for *QHD.HN-5H*, we conducted whole-genome resequencing of ‘Haruka Nijo’, ‘Nishinohoshi’, and ‘Haganemugi’ (previously identified as the likely origin of the QTL). From the total read number, we calculated the average coverage of NGS data as ×30.9 for ‘Haruka Nijo’, ×24.9 for ‘Haganemugi’, and ×35.9 for ‘Nishinohoshi’. Among the polymorphisms in the 5H QTL region (19,352,357–358,711,871 bp), 1,232 polymorphisms in 502 genes were identified between ‘Haruka Nijo’ and ‘Nishinohoshi’, through SnpEff analysis as having either “high” or “moderate” effects. Among these, we searched for those with GO terms related to flowering (Supplemental Table 4) and identified five genes (Table 3, Supplemental Fig. 4). In previous studies, HORVU.MOREX.r3.5HG0460080 was annotated as *HvTFL1* (Kikuchi *et al.* 2009) and HORVU.MOREX.r3.5HG0468460 as *HvFCA* (Kumar *et al.* 2011). The polymorphisms in four genes, namely, HORVU.MOREX.r3.5HG0446560, *HvTFL1*, HORVU.MOREX.r3.5HG0463760, and *HvFCA*, were identical in ‘Haruka Nijo’ and its ancestor ‘Haganemugi’, whereas the genotype of HORVU.MOREX.r3.5HG0427840 differed between the two cultivars.

## Discussion

### Challenges in heading date control and discovery of a previously unknown QTL in cultivars from the Kyushu region

Maximizing crop yield is critically dependent on optimizing the crop’s to the environment. In particular, heading date is a key trait that significantly influences yield and quality (Cockram *et al.* 2007). In the Kyushu region of Japan, autumn-sown barley is characterized by a very short growing season (late November to mid-to-late May) owing to its rotation with rice and the influence of the rainy season, making Kyushu a region with one of the most restricted barley growing periods worldwide (USDA 2025). Therefore, we assumed that early-heading alleles of major genes related to heading date had already been selected and fixed in barley in that region. Indeed, genotyping results for six major heading-related genes showed no differences among Kyushu’s main cultivars (Table 1), despite the fact that there are clear differences in heading date among them. In the RIL population created in this study, a stable heading date difference of 2–5 days was confirmed between the parental cultivars ‘Haruka Nijo’ and ‘Nishinohoshi’ regardless of season or test site (Fig. 1). This difference was highly reproducible regardless of temperature or sowing date (Supplemental Fig. 1, Supplemental Table 2), and a QTL with a high contribution to heading date variation was stably detected over three seasons (Table 2). *QHD.HN-5H* is located on chromosome 5H, where known heading-related genes, such as *PhyC* (527,774,453–527,779,253 bp) and *Vrn-H1* (528,147,816–528,157,990 bp), are present; however, based on genotyping results and comparisons of physical locations (Fig. 2), *QHD.HN-5H* is in a different region and we consider it to be an independent factor. These results suggest that *QHD.HN-5H* is a newly discovered key heading date control factor within the Kyushu cultivar group.

Heading date in the RIL population showed transgressive segregation, suggesting the involvement of loci in addition to *QHD.HN-5H*. Three additional QTLs were detected in our study but were not considered stable. Among them, QTLs on chromosomes 1H and 2H were detected in the 2017–2018 season, with the ‘Nishinohoshi’ allele conferring earlier heading, while a QTL on chromosome 6H was detected in two seasons (2017–2018 and 2020–2021), with the ‘Haruka Nijo’ allele conferring earlier heading. These QTLs are likely contributors to the observed transgressive segregation.

Several QTLs related to heading date have been reported in the vicinity of the 5H centromere. Alqudah *et al.* (2014) identified QTLs associated with multiple traits related to heading (e.g., spikelet differentiation, awn extraction, heading, and anther extraction) near *HvCO3*, *HvTFL1*, and *HvCMF13* on chromosome 5H in a GWAS analysis using spring-sown varieties in the *Ppd-H1* photoperiod-sensitive group. Obsa *et al.* (2016) conducted multi-season field tests in different locations in Australia using a doubled-haploid population derived from elite varieties and identified a maturity-related QTL near *HvTFL1* on chromosome 5H. Although the materials and cultivation conditions used in these studies and the present study differ, making it unclear whether the same gene was detected, these findings collectively suggest that the 5H centromere region may be an important genomic region involved in heading date control.

No non-synonymous substitutions were detected in *HvCO3* and *HvCMF13* between the ‘Haruka Nijo’ and ‘Nishinohoshi’ in this study, and prior research has shown that overexpression of *HvTFL1* in rice did not affect heading date (Kikuchi *et al.* 2009). Taken together, these findings imply that other genes, not highlighted in earlier studies, may underlie the heading date variation associated with *QHD.HN-5H*. In this study, we identified *HvTFL1* and *HvFCA*, and two other candidate genes—HORVU.MOREX.r3.5HG0446560 and HORVU.MOREX.r3.5HG0463760—based on whole-genome resequencing and GO-based functional annotation. While these genes have not been previously considered as candidate genes for heading date QTLs, their annotated functions suggest potential involvement in flowering-related processes. These results indicate that genetic components not emphasized in earlier studies may also contribute to heading date variation in barley.

### Breeding value of the chromosome 5H centromere region and pedigree-based origin analysis

The region near the centromere of chromosome 5H, where *QHD.HN-5H* is located, has been of interest in breeding because it contains the BaYMV resistance gene *rym3* (Saeki *et al.* 1999). With the emergence of BaYMV type III in the Kyushu region, the use of *rym3* began (Tsuru *et al.* 1982), and the six-rowed barley cultivar ‘Haganemugi’ from the Tohoku region was used as the donor parent for its introduction. *QHD.HN-5H* showed a detection peak near *rym3* (Fig. 2), and a strong association was observed between the *rym3* phenotype and the genotype of FB0333, the nearest marker to *QHD.HN-5H*, among the varieties and lines in the ‘Haruka Nijo’ pedigree (Fig. 4). Indeed, FB0333 (300,934,909 bp), is located near the marker used for *rym3* selection, TBr3-2 (324,742,099 bp) (Haruyama *et al.* 2012) on chromosome 5H of the ‘Morex’ genome sequence. Taken together, these observations support a likely co-introgression of the *QHD.HN-5H* region with *rym3* from ‘Haganemugi’ into Kyushu cultivars; however, this remains an inference based on phenotype and positional proximity rather than direct genotyping of *rym3*-linked markers in the pedigree lines. On the other hand, cultivars such as ‘Haganemugi’, which were developed for cold regions, are generally not used directly for breeding early-heading two-rowed cultivars; when they are used, it is mainly as sources of disease resistance rather than of earliness. Additionally, Japanese two-rowed hulled barley cultivars grown primarily in the Kyushu region are a relatively new group of cultivars based on pure-line selections from ‘Golden Melon’, which was introduced during the Meiji era (late 19th century), and these Kyushu cultivars are genetically similar (Saotome *et al.* 2009). From these findings, we conclude that, although *QHD.HN-5H* was not introduced as a direct breeding target, the chromosome region introgressed from ‘Haganemugi’ contributed to the early heading of Kyushu cultivars. This study demonstrates that genetically distant materials can contribute useful traits other than the intended breeding targets.

### Functional characterization and breeding prospects for *QHD.HN-5H*

At present, it is unclear how *QHD.HN-5H* is involved in the complex genetic pathway that controls heading. To use *QHD.HN-5H* more effectively in the future, further functional analysis is necessary to determine whether it is involved in any of the known environmental response pathways, such as vernalization requirements or photoperiodic response, or instead represents a new heading-control mechanism. *QHD.HN-5H* is located near the centromere of chromosome 5H, an area where recombination is rare, making it difficult to isolate factors using conventional recombination-based analysis. However, our WGS analysis of this region revealed five heading-related genes with non-synonymous substitutions between ‘Haruka Nijo’ and ‘Nishinohoshi’. Additionally, since *QHD.HN-5H* is linked to *rym3*, and ‘Haruka Nijo’ shares the same genotype as its ancestor ‘Haganemugi’ at *rym3* and at four of the five genes, these four have been identified as strong candidates involved in heading date differences. These candidate genes could serve as promising targets for future complementation tests and expression analyses to clarify their functions.

Early heading is generally associated with reduced yield due to shortened vegetative growth and grain filling periods. This trade-off may be particularly pronounced in the Kyushu region, which has one of the shortest growing seasons for barley cultivation worldwide. Previous studies in wheat have shown that advancing heading by one day can reduce yield by approximately 3% under the warm climate of Kyushu (Taya 1993). Nevertheless, ‘Haruka Nijo’, which carries the early-heading allele, has large grain and many spikes (Kawada *et al.* 2015), traits that likely compensate for the potential yield penalty associated with early heading. Although the effect of QHD.HN-5H on yield cannot be fully separated from other contributing factors, its presence in a high-yielding cultivar indicates that the early-heading allele can coexist with high yield and can be utilized in breeding barley adapted to short growing seasons.

Considering the possibility that *QHD.HN-5H* represents a heading date control factor distinct from previously known genes, it could serve as a target for fine-tuning heading date in future genome-based breeding efforts. Furthermore, since *QHD.HN-5H* is linked to the aforementioned resistance gene *rym3*, the use of marker FB0333 might enable simultaneous selection for early heading and disease resistance.

### Conclusion

In conclusion, the QTL identified in this study indicates the presence of previously unknown genetic diversity within the Kyushu cultivar group. Furthermore, this QTL could contribute to practical breeding strategies, such as fine-tuning heading date and simultaneous selection for disease resistance.

## Author Contribution Statement

TS conceived the research plan, conducted the experiments, and wrote the manuscript. TTon developed the RIL population. SF performed genotyping using the Dynamic Array platform. YN conducted WGS analysis. YB analyzed the data and supervised the research. TTan analyzed polymorphisms in the WGS data, contributed to the research planning, and supervised the project. JT contributed to the research planning, supervised the project, and reviewed the final manuscript. All authors read and approved the final version of the manuscript. JT is responsible for submission and communication with the journal. TTan is responsible for technical and experimental aspects. JT and TTan contributed equally and share responsibility for the manuscript as corresponding authors.

## Supplementary Material

Supplemental Figures

Supplemental Table 1

Supplemental Table 2

Supplemental Table 3

Supplemental Table 4

## Figures and Tables

**Table 1.** Genotypes of heading-date-related genes

Variety	Gene name (position)								Heading date*^c^*	
	Vernalization*^a^*				Photoperiod*^b^*				Growing season	
	*Vrn-H1* (5HL)	*Vrn-H2* (4HL)	*Vrn-H3* (7HS)		*Ppd-H1* (2HS)	*Ppd-H2* (1HL)	*PhyC* (5HL)		2016–2017 2 replications	2017–2018 3 replications
Haruka Nijo	W	S	W		E	E	E		25.0, 28.0 (26.5)*^d^*	34.3 a*^e^*
Nishinohoshi	W	S	W		E	E	E		32.0, 33.0 (32.5)	36.7 b
Harushizuku	W	S	W		E	E	E		30.0, 32.0 (31.0)	36.7 b
Sachiho Golden	W	S	W		E	E	E		26.0, 25.0 (25.5)	34.3 a

*^a^* W and S represent the winter- and spring-type alleles, respectively. *^b^* E and L indicate the early- and late-heading alleles, respectively. *^c^* The heading date is indicated considering March 1 as day 1. *^d^* For 2016–2017 (*n* = 2 plots), only descriptive statistics row data (mean) are presented, and no inferential statistics were applied. *^e^* Different letters in the 2017–2018 column indicate significant differences (Tukey’s HSD, *p* < 0.05).

**Table 2.** Summary of detected quantitative trait loci for heading date

Season	Linkage group (chromosome)	LOD	LOD peak (cM)	Nearest marker		% variance*^b^*	Additive effect*^c^*	LOD threshold
				Name	cM			
2016–2017	**LG6 (5H)** *^a^*	**14.9**	**40.0**	**FB0333**	**38.8**	**46.1**	**–1.64**	3.7
2017–2018	LG1 (1H)	7.6	49.6	FB0358	58.9	6.8	0.47	3.5
	LG2 (2H)	5.0	20.0	FB0111	16.5	7.2	0.45	
	**LG6 (5H)**	**25.5**	**39.7**	**FB0333**	**38.8**	**37.9**	**–0.97**	
	LG8 (6H)	5.8	19.5	FB0360	19.2	8.0	–0.45	
2020–2021	**LG6 (5H)**	**14.7**	**38.8**	**FB0333**	**38.8**	**30.3**	**–1.70**	3.5
	LG8 (6H)	4.7	17.5	FB0360	19.2	6.5	–0.84	

*^a^* QTL shown in bold were considered stable across environments (detected in three seasons). *^b^* Proportion of phenotypic variance explained. *^c^* Additive effect of the allele from ‘Haruka Nijo’.

**Table 3.** Summary of non-synonymous substitutions in candidate genes

Gene ID	Gene name	Physical position in 5H (bp)	Amino acid*^c^*					
			Haruka Nijo	Haganemugi	Nishinohoshi	Reference (Morex)	Residue position	Variant ID
HORVU.MOREX.r3.5HG0427840	–	19,600,546–19,601,532	K	E	E	E	139	–
			E	V	E	V	141	vcZ1GVM0
			R	R	Q	R	167	vcZ1GVLZ
			R	R	Q	R	255	vcZ1GVLX
			A	T	T	T	314	–
HORVU.MOREX.r3.5HG0446560	–	131,582,693–131,586,153	C	C	S	S	135	–
HORVU.MOREX.r3.5HG0460080	*HvTFL1^a^*	288,362,873–288,364,222	D	D	A	A	148	–
HORVU.MOREX.r3.5HG0463760	–	319,759,795–319,765,166	R	R	H	R	53	vcZ0GJ7Q
HORVU.MOREX.r3.5HG0468460	*HvFCA^b^*	353,052,076–353,062,698	Deletion	Deletion	GG	GG	63–64	–
			V	V	I	I	733	–

*^a^* Kikuchi *et al.* (2009). *^b^* Kumar *et al.* (2011). *^c^* Shaded variants are those found in ‘Haruka Nijo’.
