# Supplementary material for: QTL mapping for early heading of ‘Haruka Nijo’, the leading barley (Hordeum vulgare L.) cultivar in the Kyushu region of Japan
Source: Breed Sci. 2026 Apr 2;76(2):180–9. doi: 10.1270/jsbbs.25054 (PMC13494332; doi:10.1270/jsbbs.25054)
Supplement: Supplementary file 1 [file 76_180_s1.pdf]

doi: 10.1270/jsbbs.25054

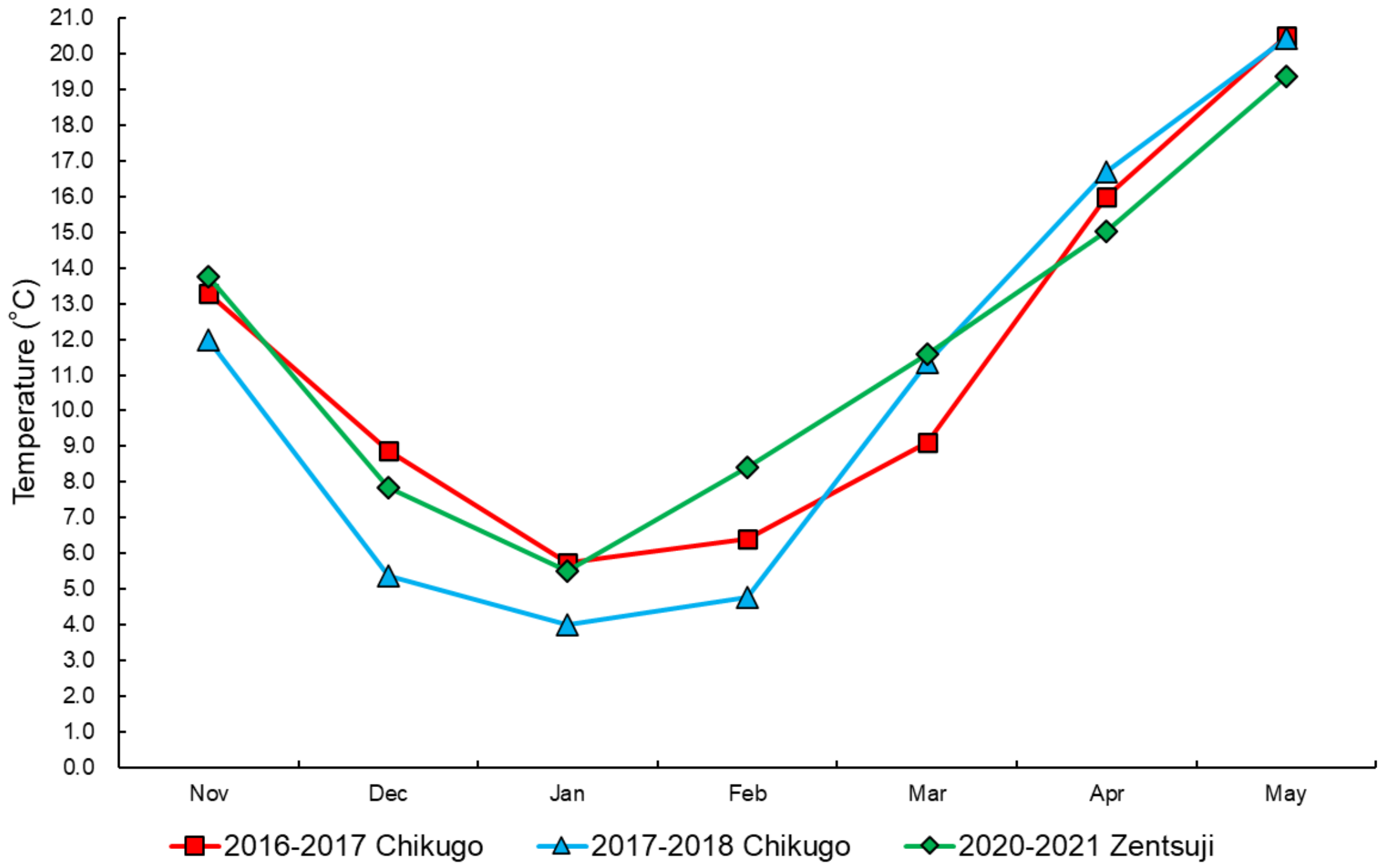

Supplemental Fig. 1. Monthly average temperature during the cultivation period (November to May).

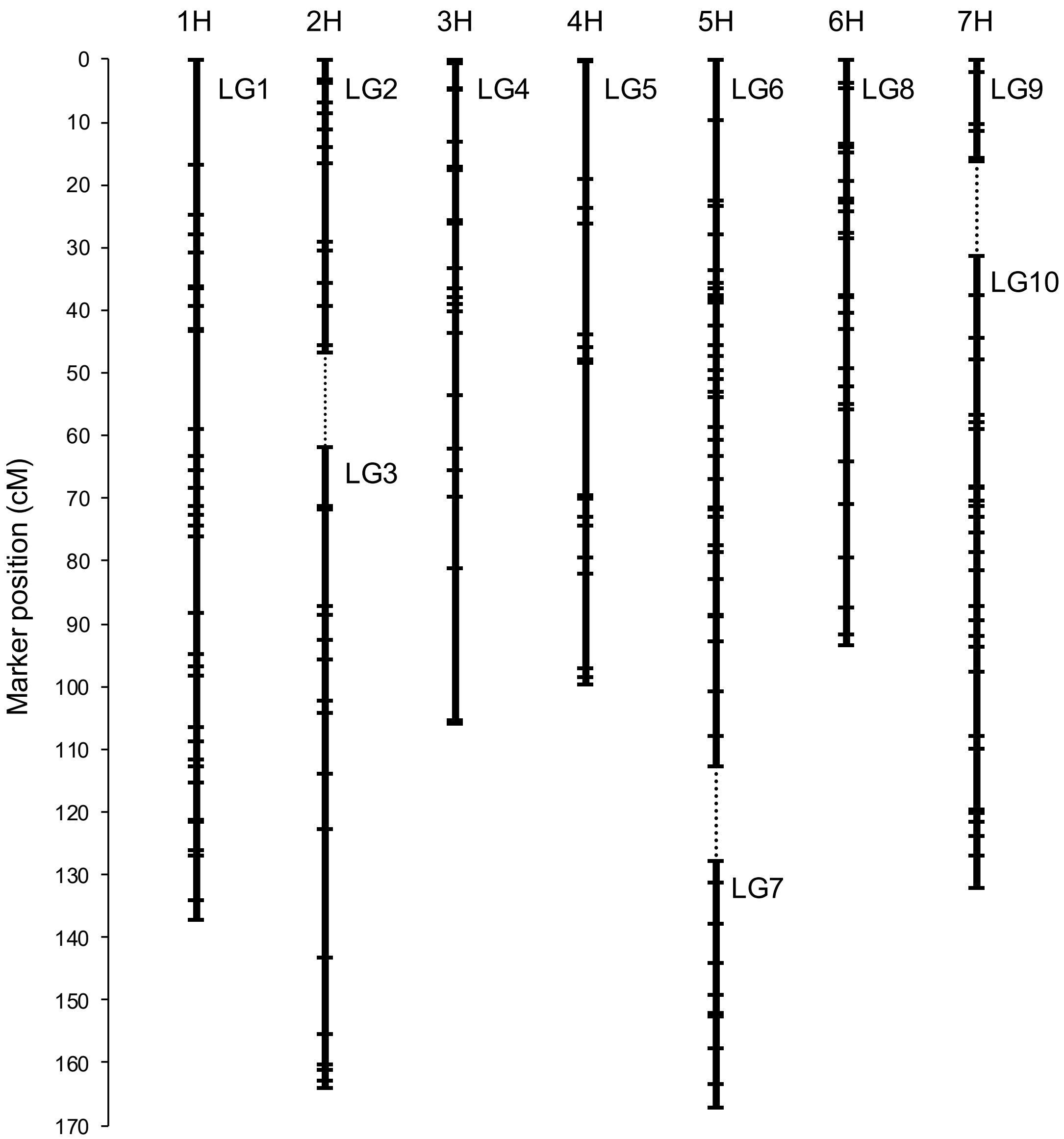

Supplemental Fig. 2. Linkage map of 'Haruka Nijo' × 'Nishinohoshi' recombinant inbred lines. Genetic distances are shown in centimorgans (cM).

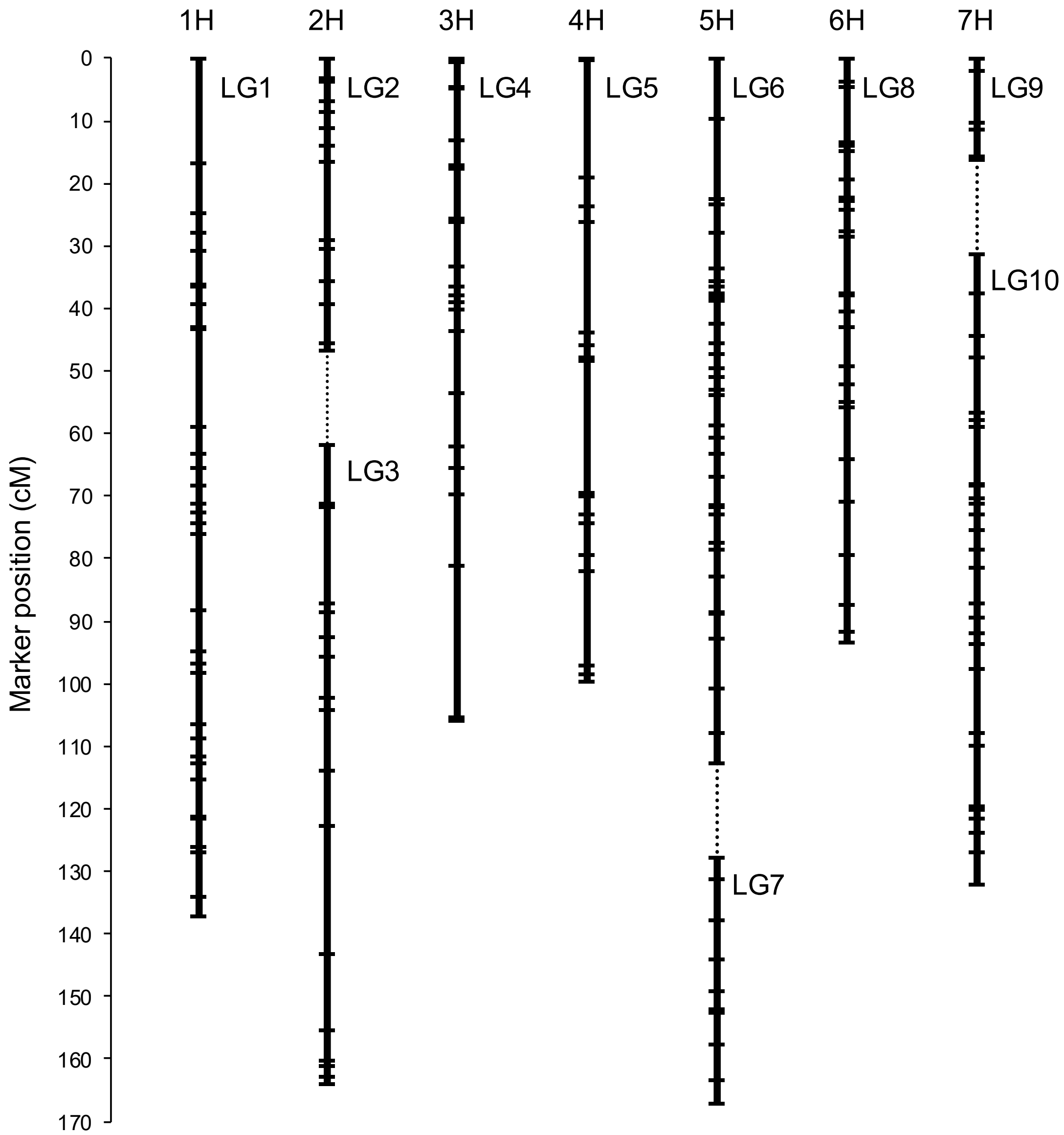

Supplemental Fig. 3. Linkage map of 'Haruka Nijo' × 'Nishinohoshi' recombinant inbred lines. Genetic distances are shown in centimorgans (cM).

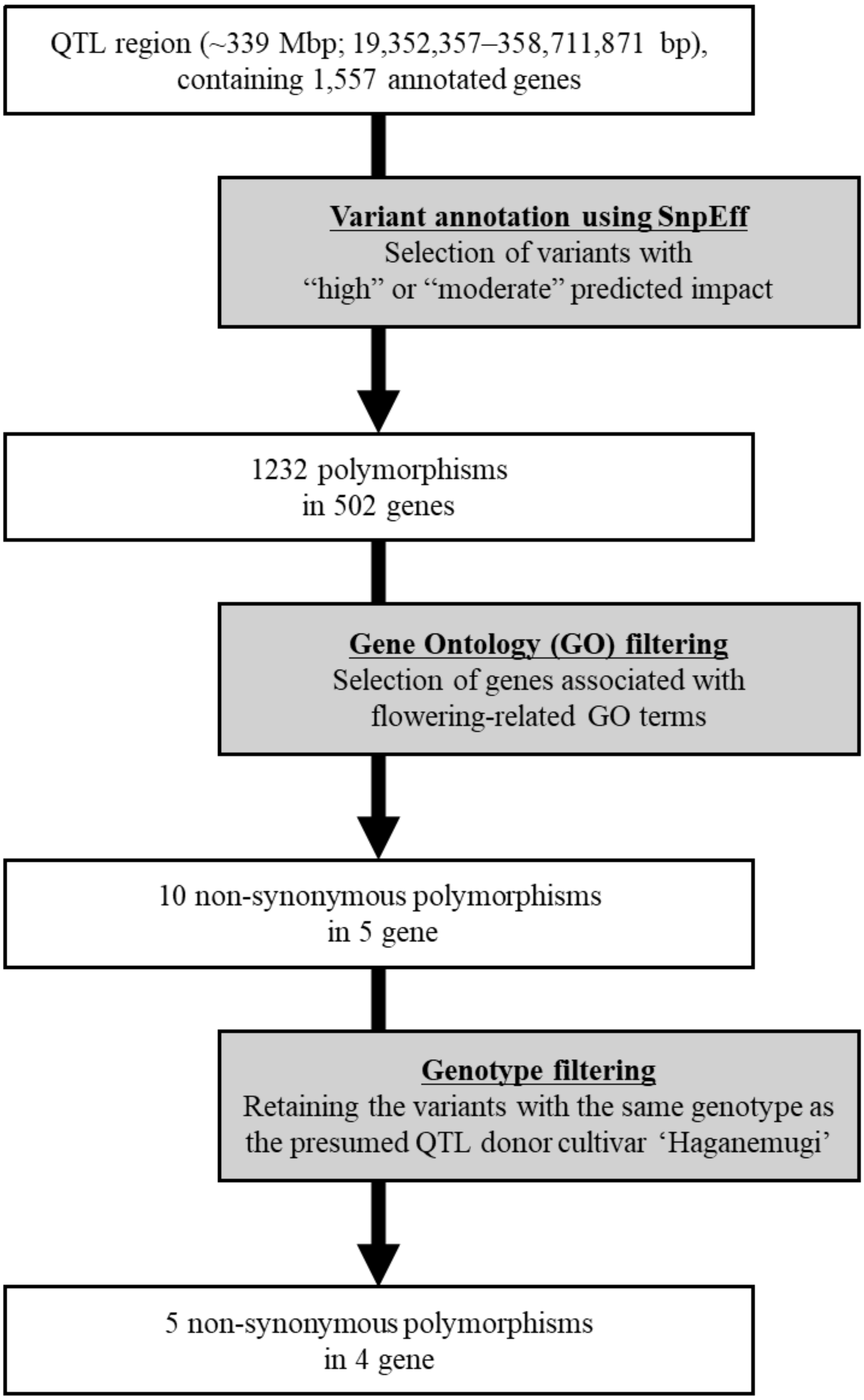

Supplemental Fig. 4. Stepwise filtering of polymorphisms within the QTL region to identify candidate flowering-related genes.
